# Extrusion Based 3D Printing of Sustainable Biocomposites from Biocarbon and Poly(trimethylene terephthalate)

**DOI:** 10.3390/molecules26144164

**Published:** 2021-07-08

**Authors:** Elizabeth Diederichs, Maisyn Picard, Boon Peng Chang, Manjusri Misra, Amar Mohanty

**Affiliations:** 1 Bioproducts Discovery and Development Centre, Department of Plant Agriculture, University of Guelph, Crop Science Building, 50 Stone Road East, Guelph, ON N1G 2W1, Canada; ediederi@uoguelph.ca (E.D.); maisyncpicard@gmail.com (M.P.); cbpchang@gmail.com (B.P.C.); 2 School of Engineering, University of Guelph, Thornbrough Building, 50 Stone Road East, Guelph, ON N1G 2W1, Canada

**Keywords:** biobased polymers, mechanical properties, thermal properties

## Abstract

Three-dimensional (3D) printing manufactures intricate computer aided designs without time and resource spent for mold creation. The rapid growth of this industry has led to its extensive use in the automotive, biomedical, and electrical industries. In this work, biobased poly(trimethylene terephthalate) (PTT) blends were combined with pyrolyzed biomass to create sustainable and novel printing materials. The *Miscanthus* biocarbon (BC), generated from pyrolysis at 650 °C, was combined with an optimized PTT blend at 5 and 10 wt % to generate filaments for extrusion 3D printing. Samples were printed and analyzed according to their thermal, mechanical, and morphological properties. Although there were no significant differences seen in the mechanical properties between the two BC composites, the optimal quantity of BC was 5 wt % based upon dimensional stability, ease of printing, and surface finish. These printable materials show great promise for implementation into customizable, non-structural components in the electrical and automotive industries.

## 1. Introduction

Three-dimensional (3D) printing, an additive manufacturing technique, is rapidly gaining popularity due to reduced material requirements and tooling time, as compared to alternative processing methods. Traditionally, computerized number control (CNC) machining has been used to make complete parts through a subtractive process. However, 3D printing offers the ability to fabricate complex geometries in an additive layer-by-layer fashion with limited to no post-print modifications [1]. Fused filament fabrication (FFF) is a relatively low-cost method of 3D printing that has extensive applications in industry, such as the biomedical [2] and aerospace [3,4] industries. FFF also functions well in rapid prototyping [5] and personal home-based printing [1]. FFF works off of the basic process of extruding a polymer-based filament through a heated nozzle to build parts layer-by-layer in the z-direction [6]. This process allows for high customization as the part shape is defined by a computer 3D model and is not limited by mold fabrication, as in the case of injection molding [7].

There are many commercial polymers commonly used for FFF. They can often be categorized as engineering thermoplastics, bioplastics, or commodity plastics [8,9,10], such as acrylonitrile butadiene styrene (ABS), polylactic acid (PLA), or high- density polyethylene (HDPE), respectively. Engineering thermoplastics show favor in many FFF applications due to superior mechanical performances and increased thermal stability as compared to commodity plastics [11]. Engineering thermoplastics can be further categorized as either biobased or petroleum based. Biobased polymers, or bioplastics, are materials that are biologically sourced, biodegradable, or a combination of both [12]. There has been a substantial drive in the industry to develop 3D printing bioplastics and engineering bioplastics.

The most commonly studied bioplastics for extrusion based 3D printing include PLA [13], polycaprolactone [14], or blends of bioplastics [15]. However, many of these bioplastics have comparatively low melting temperatures and limited mechanical performances, as compared to engineering thermoplastics [16]. To counter these limitations, engineering thermoplastics derived from organic sources are gaining popularity. One of the best examples is poly(trimethylene terephthalate) (PTT), which is partially biobased, due to one reactant being made from corn derivatives [6,17].

There is a global demand to generate products that conserve resources, recycle or refurbish products, or transform wastes into value-added products [18,19]. In the polymer industry, one of the methods to improve the sustainability of composite production is to valorize biomass waste as natural fillers. Wastes from agricultural residues [20,21], forestry residues [22], or food industries [17,23] can be diverted from landfills and instead pyrolyzed to become a carbonaceous natural filler. Pyrolysis is the thermochemical conversion of organic matter to biocarbon (BC), bio-oil, and syngas [17]. Characteristics of BC are dependent on the pyrolysis temperature, residence time, heating rate, and environmental conditions such as inert or oxygenated conditions [24]. The diversity of BC’s surface characteristics, thermal, and mechanical properties have led to its use in polymer applications in the automotive [17] and electronic industries [25]. Biocarbon can function as a composite filler suspended in a continuous polymer phase. The implementation of BC offers benefits such as reduced initial materials cost, increased thermal stability over other natural fillers, limited to no odor during processing, and increased biocontent compared to inorganic fillers [17,26,27].

The use of BC in composites is more commonly studied in injection molding practices than in 3D printing processes. However, preliminary successes have suggested BC as a potential filler in additive manufacturing applications. Idrees et al. [28] performed work to incorporate BC with recycled poly(ethylene terephthalate) PET. Significant increases in tensile properties were observed with the addition of BC. Biocarbon was successful in creating printable parts and had effects on important parameters such as the glass transition temperature (T_g_) and the coefficient of linear thermal expansion (CLTE) [28]. A reduction in CLTE was also seen with the addition of graphene into ABS, therefore increasing the thermal stability of the polymer, which is beneficial for FFF [29]. Ertane et al. [30] worked to implement BC into PLA to increase its potential applications. Interestingly, they found that FFF processing increased the interaction between the PLA matrix and the BC.

This work focuses on the implementation of BC to increase the biocontent within polymer filaments for FFF technologies. The use of a partially biobased engineering thermoplastic in addition to the natural filler was intentionally selected to offer a potential substitute for petroleum-based engineering thermoplastics with carbon black or other inorganic fillers. It is important to highlight that this work is an intermediary work. This work uses PTT derived blends containing a chain extender (CE) and impact modifier (IM) that were optimized for 3D printing dimensionally stable, complete, and warpage-free parts [6].

This work focuses on combining the aforementioned polymer blends with bioderived *Miscanthus* BC. The strategy of BC has been investigated and proven to improve the thermal stability and reduce CLTE of the polymer blends in injection molding type works. There are additional benefits to using BC, such as its use as a colorant and reduced costs from decreased quantities of expensive polymer required. Similar principles applied and the content of BC was optimized for printing complete and warpage-free parts. The goal of this work was to create novel printing materials that could be used in big-area-additive manufacturing. This PTT blend with the addition of BC has potential for use as 3D printing composite materials for non-structural automotive parts and electrical components.

## 2. Results and Discussion

### 2.1. Characterization of Biocarbon

**Figure 1:**
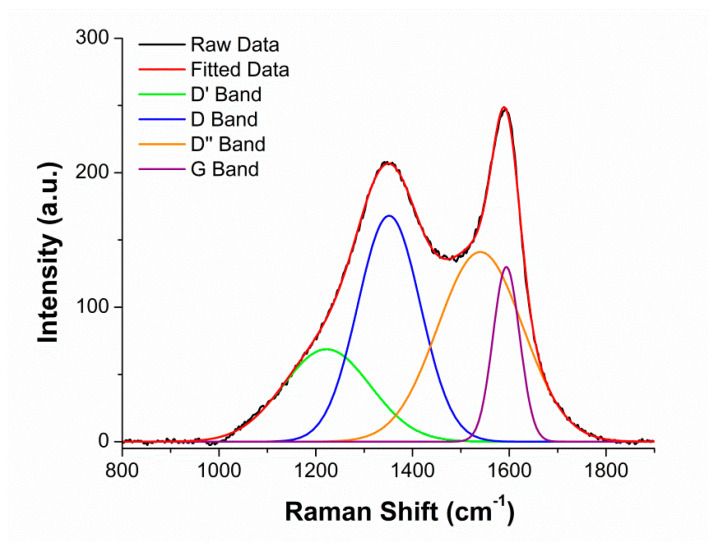
Raman spectra of *Miscanthus* BC pyrolyzed at 650 °C and after 24 h of ball milling.

The surface characteristics and general morphological shapes of the BC were determined via Raman spectroscopic analysis (Figure 1). The Raman analysis compared the graphitic content in the G band to the disordered content in the D band. The D and G peaks of BC reached maximal values at 1350 and 1589 cm^−^^1^, respectively. These peaks corresponded with similar peaks reported in the literature for other BC samples [25]. A ratio of intensities from the D/G bands was 1.3. Graphene aerogels with a similar intensity ratio have also found success in 3D printing [31]. This ratio suggests that there is more disordered content than organized carbon. The difference in peaks is a result of in-plane vibrations associated with sp^2^ carbon in graphite lattices and plane stretching [32].

Based on the SEM analysis of the same *Miscanthus* BC studied by our lab [20], it was determined that the ball milling caused the BC to have a more uniform shape and smaller size distribution [20]. This *Miscanthus* BC with 24 h of ball milling was studied previously and found to have an average particle size of 0.9 μm [20]. Since the size of the particle determines the precision of the print [33], it was important to have small particles. The smaller sized filler was also used to reduce potential clogging at the nozzle. The next steps for this research would be to determine the correlation between graphitic structure and intrinsic properties connecting Raman spectroscopy and surface morphology through X-ray diffraction (XRD) analysis.

*Miscanthus* BC at 650 °C was chosen since lignocellulosic biomass derived BC offers a higher modulus than other sources of BC due to a high stiffness of samples. The increased stiffness is further associated with increased polymer performance [21]. Since the BC was pyrolyzed at a lower temperature, the BC was able to maintain more surface functionalities than samples pyrolyzed at higher temperatures [17]. These surface functionalities on the BC interact with the functional polymers in the blend [34].

### 2.2. 3D Printed Composites

The 3D printed composites are referenced as blend content/BC content. The blend is comprised of 90 wt % PTT, 10 wt % IM and 0.5 phr of CE. Further details can be found in Table 2 below as well as the materials section.

The challenges and analyses found of 3D printing with the BC composites are outlined in the sections below. A preliminary investigation on the mechanical properties were performed and reported with injection molded composites with 0, 2.5, 5, 7.5, and 10 wt % BC (refer Table S1, in the Supplementary Materials). Minimal differences were seen in flexural and tensile properties of the BC composites, therefore 5 and 10 wt % BC compositions were chosen for further testing with 3D printing.

#### 2.2.1. Challenges of Biocarbon in Printing

**Figure 2:**
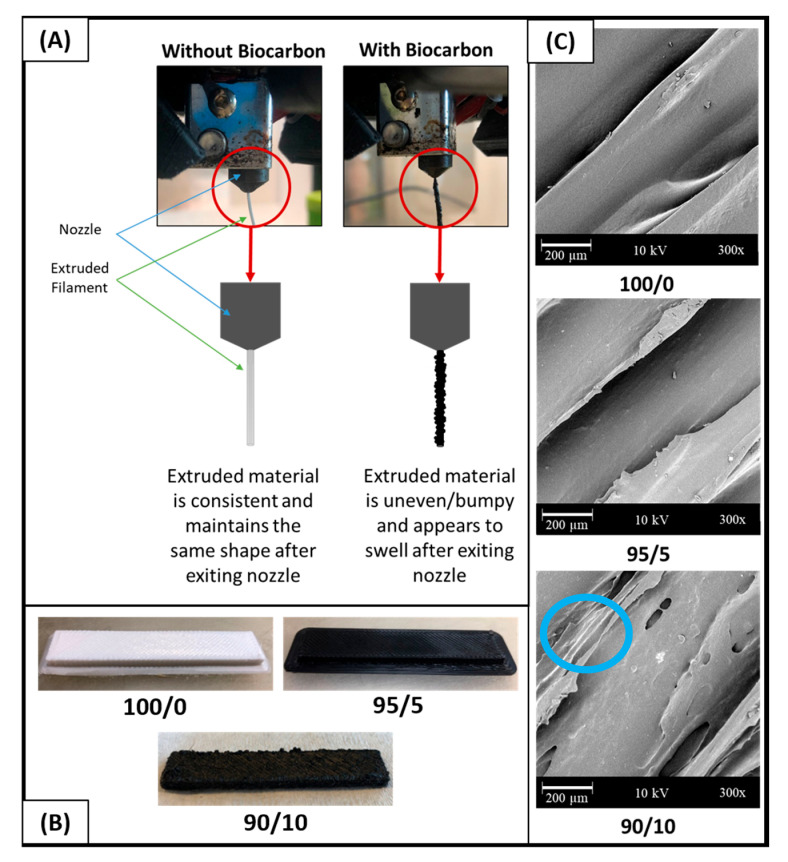
PTT/BC biocomposites in FFF: (**A**) the upper left section provides a comparison of an extrudate with and without biocarbon to depict challenges with consistency, (**B**) the lower left section compares FFF samples with different BC content for surface roughness, and (**C**) the right side of the image examines SEM images of the FFF surface of FFF printed samples. See Table 2 for nomenclature.

Printing composites with BC can be challenging; for example, various works have reported the degradation of the printer nozzle due to the abrasiveness of BC [30]. Jamming at high BC loadings and poor surface finishes have also been reported [30]. The challenges with interlay adhesion and due to the addition of a filler were also examined and are discussed in Section 2.2.6 below. Figure 2A depicts the extrudate from the FFF printed nozzle comparing the polymer blend to that of the composites. To further clarify, a schematic was drawn to highlight that filaments containing BC tended to be less consistent and had a rougher appearance. The slight swelling seen after the material exits the nozzle could have been responsible for the rougher, poorer quality surface finishes on the higher BC content FFF parts, as seen in Figure 2B. The 100/0 and 95/5 samples displayed smooth, cleanly defined rasters; however, the 90/10 printed samples were very rough and contained clumps of polymer and BC on the surface. This is represented in lower left section of the image below (Figure 2B) for the polymer blend and composites containing 5 and 10 wt % of BC. To examine the aforementioned parts on a microscopic level, SEM surface images were captured at a 300 times magnification (Figure 2B). Voids, identified by the blue circle on the Figure 2C, were most prominent on the surface of the 90/10 samples. Ertane et al. [30] also observed an increase in surface voids with increased BC content. The changes in surface finish of the BC composite materials likely relate to the rougher surface finish of the *Miscanthus* fiber. This is more prominent at the 10 wt % fiber since more fibers are present overall. The challenges and impacts of the natural filler surface and its correlation to print quality and mechanical performance are summarized by Duigou et al. [35] from combining literary works. Although challenges exist with the inclusion of BC into FFF feedstocks, low additions of BC did not decrease the printability of the filament. From its effects on printability and surface finish, 5 wt % biocarbon content is optimal as comparable to 10 wt %.

#### 2.2.2. Thermogravimetric Analysis (TGA)

**Figure 3:**
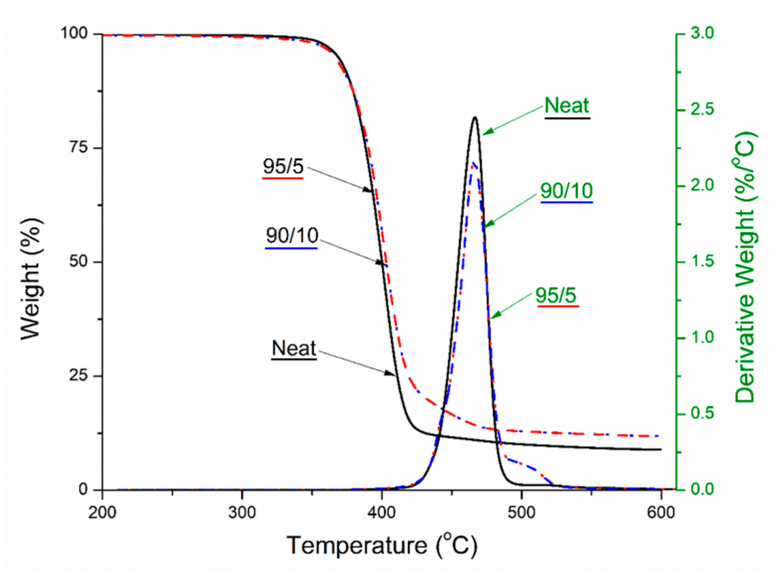
Thermogravimetric analysis (TGA) curves (weight and derivative weight) of the PTT blends and its biocomposites. See Table 2 for nomenclature.

TGA analysis is important for looking at the degradation characteristics of a material and the thermal stability over a range of temperatures. The maximal degradation temperature of PTT was about 350 °C [36,37]. There was no significant shifting in the maximal degradation temperature peak, but there was an improvement in the thermal stability as can be seen clearly in the TGA curves. The increase in thermal stability was noted from the curve relating to the change in weight (%) as there were less losses over the higher temperatures (Figure 3). The derivative curves highlight the presence of BC through the elevated section at 500 °C. This again is associated with reduction in degradation at this temperature. When BC is pyrolyzed at temperatures greater than that of the maximal polymer degradation temperature, BC offers increased thermal stability to the composites [38]. The improvement in thermal stability was further confirmed in the CLTE analysis. This is critical for higher temperature applications such as internal automotive components that operate at higher temperatures.

#### 2.2.3. Thermomechanical Analysis (TMA)

**Figure 4:**
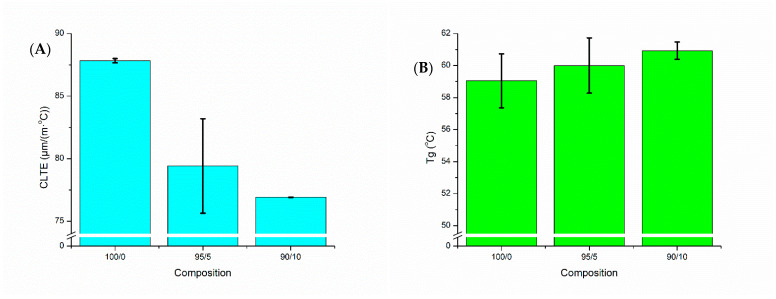
Thermomechanical analysis of the blend and composites: (**A**) coefficient of linear thermal expansion (CLTE) and (**B**) glass transition temperature (T_g_) where injection molded samples were used. See Table 2 for nomenclature.

CLTE and the T_g_ are important properties to investigate for success in FFF. CLTE is a measure of the expansion of the material at elevated temperatures, which can be used to approximate the swelling or shrinking that could occur during 3D printing processes. In a study by Fitzharris et al. [39], CLTE was found to have a substantial impact on the success of printing due to its contributions to warpage and reduced thermal stability. A direct correlation was observed between CLTE and warpage, and many studies have shown that fillers tend to decrease CLTE [21,27,39]. Therefore, it is important to note that the addition of BC into PTT decreased the CLTE, as seen in Figure 4A. The lowest CLTE was seen in the 90/10 composite, at a value of 13% less than the 100/0 value. CLTE decreases with filler addition due to their effect of hindering polymer chain movement [40]. T_g_ is also important to FFF as it dictates what build platform (or bed) temperature range will be successful. A bed temperature slightly above T_g_ has been found to be optimal in a study by Spoerk et al. [41]. Insignificant changes in T_g_ were seen due to the influence of BC, as shown in Figure 4B. The polymer blend and composites had a T_g_ over the range of 59–61 °C. This is increased from neat PTT’s T_g_ of 45–55 °C [42], which indicated the additives and BC had an effect on the neat PTT’s thermal properties. Printing of these composites was done with bed temperatures ranging from 55 °C in the 100/0 to 80 °C in the 90/10. Bed temperatures were kept similar to the T_g_, however increasing BC content in the samples resulted in a necessary increase in bed temperature to ensure good bed adhesion. 

#### 2.2.4. Rheology

**Figure 5:**
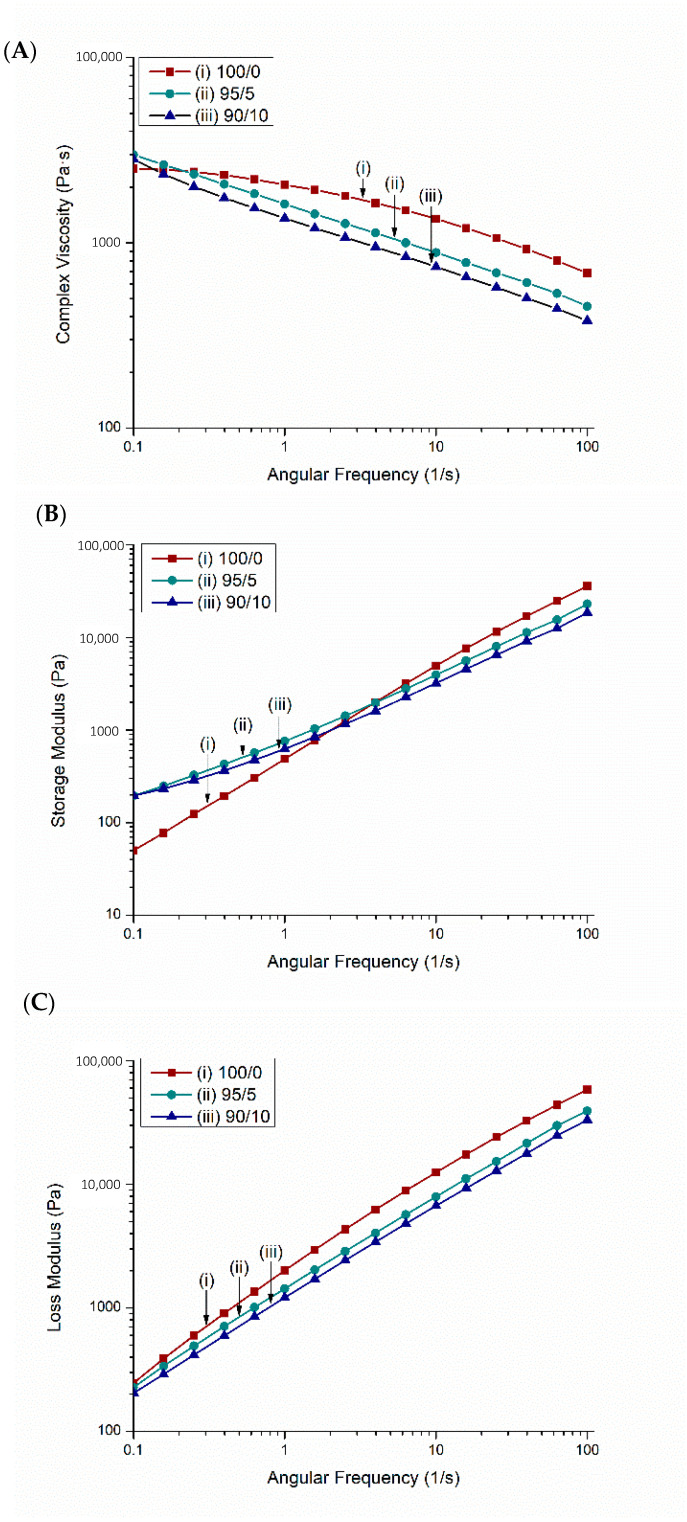
Rheological analysis of composites: (**A**) complex viscosity (η), (**B**) storage modulus (G′), and (**C**) loss modulus (G″) for injection molded samples of the PTT blend and *Miscanthus* BC composites. See Table 2 for nomenclature.

The three rheological properties of the PTT blend and its biocomposites were determined from rheological analysis including complex viscosity (Figure 5A), storage modulus (Figure 5B), and loss modulus (Figure 5C). The complex viscosity of the PTT blend decreased as the angular frequency increased, which showing a typical shear thinning behavior. The complex viscosity was found to decrease after incorporation of BC and further decreased as the BC content increased from 5 to 10 wt %. Similar results were found with engineering thermoplastic polyetheretherketone (PEEK) and carbon fibers [43]. This observation suggests that the composites are more sensitive to high shearing action [6]. Nguyen et al. [44] describe the ideal zone for 3D printing composites; based on their research, the challenges with printing the 90/10 composites were a result of too low of a complex viscosity [44]. The shear thinning behavior of the material can be used to determine the required pressure and force to extrude the molten polymer through the FFF nozzle [45].

The storage modulus defines the potential energy stored in the materials and thus describes its elastic response under deformation [46]. The PTT blend and its biocomposites were considered to be frequency dependent since the storage modulus and loss modulus value increases with increasing frequency. This has also been found in other composites [46].

#### 2.2.5. FFF Mechanical Properties

The mechanical performances of the FFF printed samples are presented in Table 1. As compared to the blend (100/0), the 95/5 and 90/10 composites both experienced a loss in mechanical performance. There is not a significant difference in the mechanical performance between the 5 wt % and 10 wt % BC. The reduction in mechanical performance was attributed to poor layer adhesion and agglomeration of particles, as noted in SEM images below (Figure 5). The printing parameters were optimized through systematic trials to produce parts that were free of warpage and delamination. Poor layer adhesion was exacerbated by limitations with the FFF printer used, such as the lack of an environmental temperature control chamber. Furthermore, it was likely that the agglomeration of particles at the nozzle of FFF printers, also discussed by Zhang et al. [47], resulted in poor flow of the materials. Further works with large scale additive manufacturing technologies with heated chambers will be investigated as it is anticipated that there would be improvements in the mechanical performance of such parts. For the current work, it is recommended that materials be used for non-structural components in FFF as there are some limitations with the mechanical performances. An example of non-structural parts would be interior car parts that are non-load bearing.

#### 2.2.6. Scanning Electron Microscopy 

**Figure 6:**
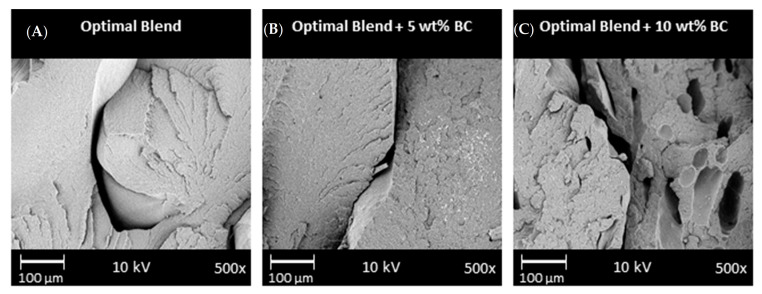
SEM images of the impact fracture surface for FFF samples for the (**A**) optimal polymer blend, (**B**) optimal blend with 5 wt % biocarbon, and (**C**) optimal blend with 10 wt % biocarbon. See Table 2 for nomenclature for optimal blend.

Micrographs, taken via SEM, show that all the FFF samples experienced poor layer adhesion, noted by the voids and gaps in the samples (Figure 6). There were substantially more noticeable voids in the 10 wt % BC samples, as noted by the blue circles on Figure 5C. Similar voids have been found when PLA and BC composites were 3D printed [30]. The pullout voids were likely one aspect that led to the decreases in mechanical performance. In work with other 3D printed composites, researchers have suggested that the extrudate is only heated to a semi-molten state rather than completely molten state, which has reduced the fusion of layers before cooling [48]. It is likely these same phenomena were seen in this work due to the presence of BC and with limitations of the nozzle temperature.

One of the goals of this project was to determine which composite offered superior performance. Superior performance was determined via comparisons of the surface finish, mechanical performance, CLTE data, and the SEM morphological analysis of the layers for both the 5 and 10 wt %. The ideal content of BC would be suggested at 5 wt % since this content maintains a smooth surface finish and offers comparable mechanical performance and CLTE to 10 wt % BC. Printing samples with 5 wt % BC are superior at maintaining dimensional accuracy. With higher BC content than 10 wt %, samples had extremely poor printability with this FFF printer configuration.

## 3. Materials and Methods

### 3.1. Materials

Sorona^®^ PTT is a biobased engineering thermoplastic and was sourced from DuPont (Wilmington, DE, USA). It has a 37% biobased content that comes from corn-derived 1,3-propanediol [31]. The chain extender (CE) used was poly(styrene-acrylic-co-glycidyl methacrylate), known as Joncryl 4368, and was purchased from BASF (Ludwigshafen, Germany). An impact modifier (IM), poly(ethylene-n-butylene-acrylate-co-glycidyl methacrylate) trademarked Elvaloy PTW, was also incorporated. PTT, the CE and the IM composed the matrix of the composites. The biocarbon was provided by Competitive Green Technologies (Leamington, ON, Canada). The BC, made from *Miscanthus*, was pyrolyzed at 650 °C and then ball milled for 24 h. This BC was then dried in an oven at 100 °C until the moisture was less than 0.2% before compounding. A Sartorius infrared moisture analyzer (Gottingen, Germany) was used to confirm the moisture content.

### 3.2. Characterization of Biocarbon

#### Raman Spectroscopic Analysis

To determine the relative concentrations of disordered carbon versus ordered carbon, Raman spectroscopic analysis was performed. A Thermo Fisher Scientific DXR^TM^ 2 Raman microscope (Waltham, MA, USA) was used at a magnification of 10 times. A 532 nm laser at 5 mW power was operated through a 50 μm pinhole to collect data over the range of 50–3000 cm^−1^. For peak deconvolution, four peaks were used in combination with the Gaussian–Lorentzian area mode as this is common practice in the literature [49].

### 3.3. Processing Methods

#### 3.3.1. Reactive Extrusion

Pellets of PTT were dried at 80 °C for 24 h (moisture content <0.2%) and were mixed by hand with the CE, IM, and dried BC. This was then fed into a Liestritz-Micro-27 co-rotating twin screw extruder (Nuremberg, Germany) and pellets and filaments were collected. Materials were processed at 240 °C with a 100 rpm screw speed and a 7 kg/h feed rate. The processing conditions were established from previous work [6].

#### 3.3.2. Injection Molding

Pellets with a moisture content of <0.2% were fed into an Arburg AllRounder 77 Ton (Loßburg, Germany) coinjection molding machine. Processing settings were a 240 °C barrel temperature, 80 °C mold temperature, and a 20 s hold time. Samples were created for thermomechanical and rheological analyses. 

#### 3.3.3. 3D Printing

Samples were 3D printed using FFF technology on a Lulzbot Taz 6 (Fargo, ND, USA) printer system. The 3D models were generated using SolidWorks (Dassault Systems, Vélizy-Villacoublay, France) and the printer was run using the Cura Lulzbot Edition (2.6.69) software (Ultimaker, Geldermalsen, The Netherlands). A 0.5 mm diameter brass nozzle and a borosilicate glass/polythyleneimine bed were used. The implemented printing parameters were 65 °C bed temperature, 280 °C nozzle temperature, 0.3 mm layer height, and 35 mm/s print speed.

### 3.4. Nomenclature and Composition

The matrix used for the composites in this study was a blend of PTT, CE, and IM. The blend composition used was the optimal composition found in work done on printing PTT by Diederichs et al. [6]. This optimal blend was 90 wt %, 10 wt %, and 0.5 phr of the PTT, IM, and CE, respectfully. The blend was then combined with *Miscanthus* BC at varying weight percentages. The nomenclature and compositions of the composites can be found in Table 2.

The amount of BC incorporated into the blends was based on previous research. In another work with an engineering thermoplastic polyester, PET, authors had success in printing samples with BC loadings from 0.5 to 5% [28]. Incremental increases in BC were performed to determine a maximal print content at 10 wt % for the printer configuration used in this work. Further details are described below.

### 3.5. Characterization

#### 3.5.1. Mechanical Testing

Flexural and tensile properties were tested on an Instron Universal Testing Machine model 3382 (Norwood, MA, USA). The crosshead speed for the tensile test was 5 mm/min, while the crosshead speed for the flexural test was 14 mm/min that is analogous to ASTM D368 (type IV) and ASTM D790, respectively. Izod notched impact testing was performed on a Zwick/Roell HIT25P impact tester (Ulm, Germany) in accordance to ASTM D256.

#### 3.5.2. Differential Scanning Calorimetry

A heat–cool–heat analysis was performed on a TA Instruments DSC Q200 (New Castle, DE, USA) with a nitrogen flow rate of 50 mL/min from 0 to 250 °C at a rate of 10 °C/min. From this, the heat of fusion, melting temperature, and degree of crystallinity were analyzed. Equation (1) was used to calculate the degree of crystallinity 1:

(1) $$Degree\;of\;Crystalinity=\frac{∆H_{m}}{W_{f,\;PTT}\times ∆H_{m}^{o}}\;$$

where ∆*H_m_* is the heat of fusion of the sample, *W_f_* is the weight fraction of *PTT* in the composite, and $∆H_{m}^{o}$ is the heat of fusion for 100% crystalline *PTT*, which is 30 kJ/mol [50]. The 0.9, 0.81, and 0.855 were the *W_f_* values for the neat polymer blend, 10 wt % BC, and 5 wt % BC, respectfully. The DSC section is included in the Supplementary Information.

#### 3.5.3. Thermogravimetric Analysis 

A TGA Q500 (TA Instruments, New Castle, DE, USA) was used to analyze the degradation temperature of the composites. The samples were heated to 500 °C at a rate of 10 °C/min in a nitrogen environment. 

#### 3.5.4. Thermomechanical Analysis 

A TMA Q400 (TA Instruments, New Castle, DE, USA) was used to analyze the glass transition temperature (T_g_) and coefficient of linear thermal expansion (*CLTE*) of the injection molded samples in the flow direction. The thermal history was removed from the samples by heating to 70 °C and cooled prior to recording data. The samples were then heated from −60 to 170 °C at a rate of 5 °C/min. A force of 0.05 N was applied to the sample. The *CLTE* was recorded before T_g_ in the range of −30–40 °C. The formula for calculating the *CLTE* can be found in Equation (2) below:

(2) $$CLTE=\;\frac{\frac{∆L}{L_{o}}}{\Delta T}\;$$

where Δ*L* is the change of sample length, *L_o_* is the original length of the sample, and Δ*T* is the change in temperature.

A DMA Q800 (TA Instruments, New Castle, DE, USA) was used to analyze the heat deflection temperatures (HDTs) of the composites. The samples were heated from 0 to 180 °C and the reported temperature is when the samples reached a deflection of 250 µm, in accordance with ASTM D648.

#### 3.5.5. Rheology

A Modular Compact Rheometer 302 manufactured by Anton Paar (Graz, Austria) was used to analyze the viscoelastic properties of the composites. Complex viscosity (η), storage modulus (G′), and loss modulus (G″) were tested at 250 °C using a frequency sweep test from 0.1 to 100 rad/s. Rheological properties determine how the materials behaves under a given force. It has been suggested in the literature that rheological studies of 3D printing materials can lead to successful printing and identification of printable materials [51].

#### 3.5.6. SEM of Composites

A Phenom-world ProX SEM (Eindhoven, The Netherlands) was used to image the fractured Izod impact surfaces and the FFF surface finish of the samples. This was also used to analyze the surface of the BC. The SEM images were taken at 10 kV and variable magnifications. Prior to imaging, samples were gold-coated for 10 s with a Cressington Sputter Coater 108 (Watford, England) auto vacuum sputter chamber.

## 4. Conclusions

To increase the use of FFF in industries such as the automotive and electrical, there is demand for thermally stable, sustainable printing materials. Such materials could be used in customizable jig and fixtures for product assembly and non-structural components or electrical housings. This work is a short communication on the adaption of PTT blends from previous work and their combination of *Miscanthus* BC for extrusion-based 3D printing. The PTT blend when combined with 5 wt % BC was tougher than that of 10 wt % BC and was able to maintain better dimensional accuracy during the print, therefore leaving a more visually appealing print. Further benefits of this new combination of materials were the reduction in CLTE and increased glass transition temperature over that of the PTT blend. This is more favorable for higher temperature applications, especially for use in big area additive manufacturing equipment where the thermal exposure to materials is greater. The combination of materials and printing parameters resulted in successful production of complete and warpage free samples in small scale extrusion 3D printing. The samples could be further studied for their potential in commercialized filament. The goal of increasing biocontent in engineering thermoplastics was achieved. In due time, materials like these PTT biocomposites will lead the way in sustainable product development in industries like the automotive, aerospace, and electronics industries.

## Figures and Tables

**Table 1:** Mechanical properties of FFF samples.

Sample Composition	Tensile Strength (MPa)	Tensile Modulus (GPa)	Elongation at Break (%)	Impact Strength (J/m)
100/0	35.3 ± 2.41	1.77 ± 0.10	6.34 ± 0.79	61.26 ± 11.87
95/5	26.4 ± 3.70	1.31 ± 0.22	4.01 ± 0.78	34.05 ± 4.71
90/10	28.3 ± 1.09	1.49 ± 0.09	3.54 ± 0.53	32.25 ± 3.85

**Table 2:** Nomenclature and composition of composites.

Composition of Samples	Blend * Content (wt %)	Biocarbon Content (wt %)
100/0	100	-
95/5	95	5
90/10	90	10

* Blend: 90 wt % PTT, 10 wt % IM, and 0.5 phr CE.

## Data Availability

The data presented in this study are available in Supplementary Material.

## Supplementary Materials

The following are available online, Table S1: Mechanical properties of injection moulded composites.

## Acronyms

ABS	Acrylonitrile butadiene styrene
BC	Biocarbon
CE	Chain extender
CLTE	Coefficient of linear thermal expansion
CNC	Computer numbered control
DSC DMA	Differential scanning calorimetry Dynamic mechanical analysis
FFF	Fused filament fabrication
HDT	Heat deflection temperature
IM	Impact modifier
PEEK	Polyetheretherketone
PET	Poly(ethylene terephthalate)
PLA	Poly(lactic acid)
PTT	Poly(trimethylene terephthalate)
SEM	Scanning electron microscopy
TGA	Thermogravimetric analysis
XRD	X-ray diffraction
3D	Three-dimensional
