# Supplementary material for: Extrusion Based 3D Printing of Sustainable Biocomposites from Biocarbon and Poly(trimethylene terephthalate)
Source: Molecules. 2021 Jul 8;26(14):4164. doi: 10.3390/molecules26144164 (PMC8305183; doi:10.3390/molecules26144164)
Supplement: Supplementary file 1 [file molecules-26-04164-s001.zip › molecules-1219894-supplementary.pdf]

Supplementary

Table S1 – Mechanical properties of injection moulded composites.

Composite	Tensile Strength (MPa)	Tensile Modulus (GPa)	Flexural Strength (MPa)	Flexural Modulus (GPa)	Impact Strength (J/m)
100/0	55.7 ± 0.36	2.313 ± 0.071	84.95 ± 0.46	2.293 ± 0.007	97.65 ± 4.07
97.5/2.5	59.5 ± 1.32	2.249 ± 0.025	88.93 ± 1.03	2.417 ± 0.041	69.60 ± 5.23
95/5	57.7 ± 0.41	2.453 ± 0.096	86.83 ± 0.67	2.409 ± 0.011	58.30 ± 7.33
92.5/7.5	58.7 ± 0.70	2.325 ± 0.045	88.24 ± 0.68	2.491 ± 0.022	51.78 ± 3.09
90/10	57.7 ± 0.87	2.547 ± 0.045	88.62 ± 0.90	2.536 ± 0.026	57.78 ± 5.92
